# Jugular Vein Insufficiency and Choroidal Neovascularization in Moderate Myopia: A New Unknown Factor of Additional Risk?

**DOI:** 10.1155/2015/960950

**Published:** 2015-04-21

**Authors:** Massimiliano Farina, Cristiano Ratti, Eugenio Novelli

**Affiliations:** ^1^Phlebolymphology Center, Monza Polyclinic, 20900 Monza, Italy; ^2^Ophthalmology Center, Clinical Institutes “Milan City Studies”, 20131 Milan, Italy; ^3^Biostatistics Unit, San Gaudenzio Clinic (Monza Polyclinic Group), 28100 Novara, Italy

## Abstract

To date, choroidal blood flow reduction in highly myopic eyes appears to be related to the development of choroidal neovascularization secondary to local reduction of arterial flow. Instead, no evidence of choroidal neovascularization was found in subjects with low or moderate myopia. The authors' aim has been to encourage new studies regarding the potential role of chronic retinal venous congestion in the pathogenesis of choroidal neovascularization. In December 2011, a 54-year-old woman with moderate bilateral myopia had a sudden block upon swallowing while she was eating. Subsequently (January 2013) metamorphopsia in the left eye revealed macular degeneration with choroidal neovascularization. The related echo-color Doppler study of the neck veins, performed in November 2014, showed an atypical left jugular insufficiency associated with homolateral hypertension of the superior ophthalmic veins. This singular case highlights the necessity to further investigate the potential role of chronic alterations of intra- and extracranial venous drainage in the disruption of choroidal flow in myopic patients.

## 1. Introduction

Maculopathy is one of the most important complications of myopia; the severity of this condition is associated with increased axial length and increased age [1]. Likewise, the prevalence increases with age and increasing myopic refractive error [2]. Laser Doppler velocimetry and echo-color Doppler (ECD) studies in humans and animals suggest that choroidal blood flow reduction in highly myopic eyes appears to be related to the development of choroidal neovascularization (CNV) [3, 4]. No studies on the role of retinal chronic venous hypertension in the disruption of choroidal flow are available. Here, we report on a patient with moderate myopia in which the left CNV is associated with homolateral jugular vein insufficiency.

## 2. Case Presentation

In December 2011 a 54-year-old woman, suffering from a bilateral spherical equivalent refraction of −5.2 diopters, experienced a violent pain in the jugular notch that was transmitted to the head, after an episode of a sudden block upon swallowing, while she was eating. Since this episode, she experiences pain while coughing and generally with every Valsalva maneuver. In January 2013, metamorphopsia in the left eye revealed macular degeneration with CNV.  Table 1 shows her ophthalmological examination.

**Figure 1:**
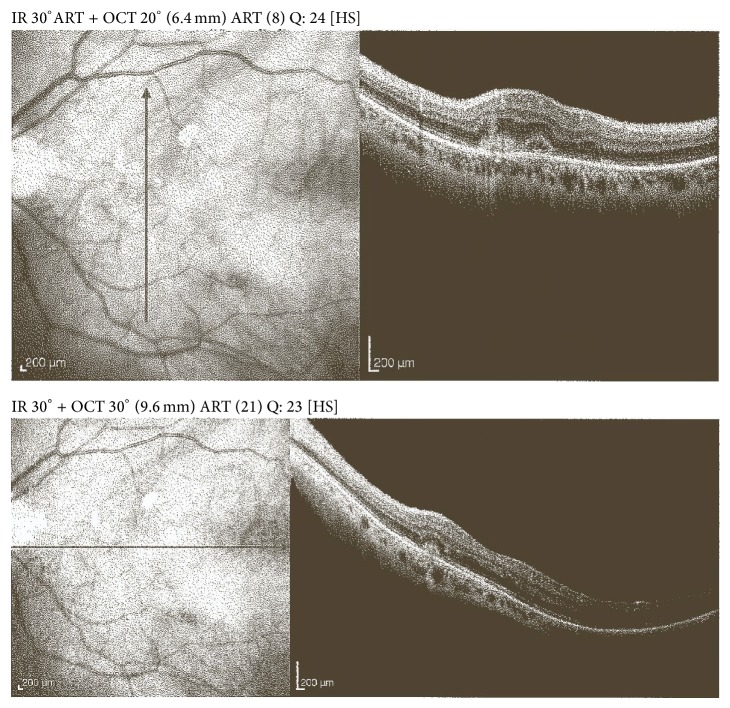
Retinal degeneration shortly after the onset of the hemorrhage.

**Figure 2:**
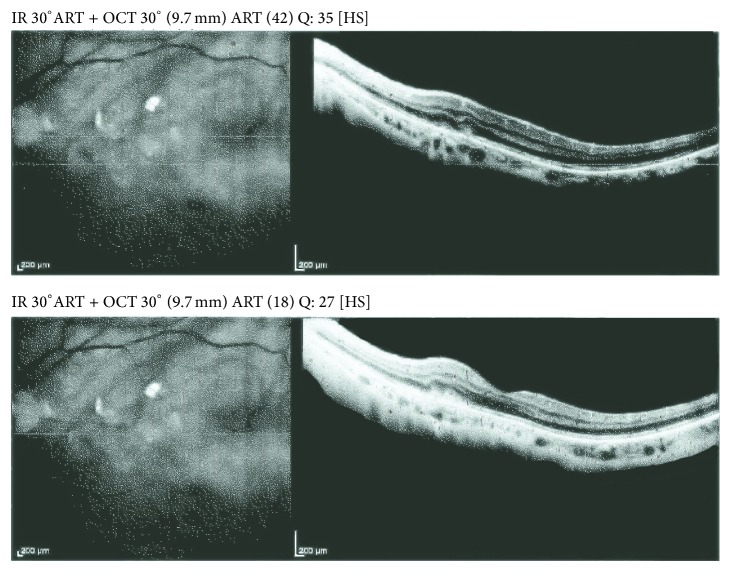
Evolution after hemorrhage reabsorption.

Optical coherence tomography (OCT) best describes the situation (Figures 1 and 2).

**Figure 3:**
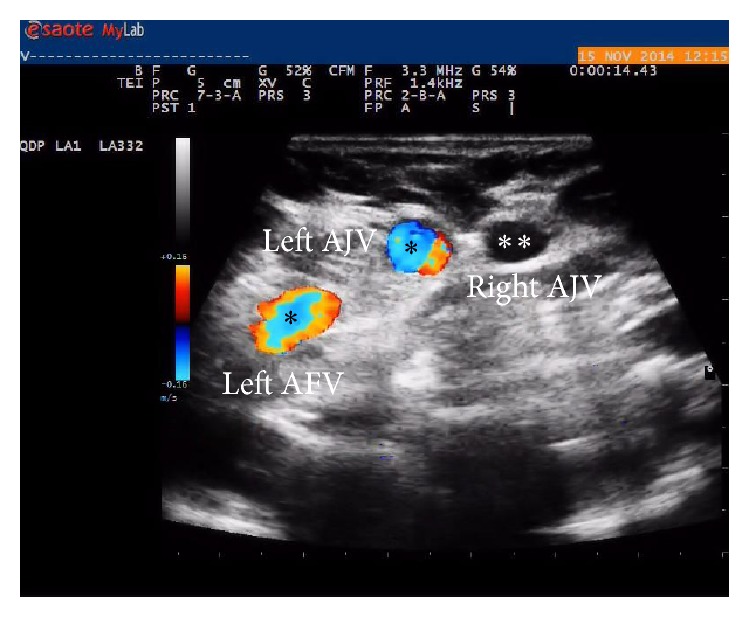
Normally evoked blocked flow (∗∗) in the right anterior jugular vein (AJV) with abnormal reflux (∗) in the anterior jugular (AJV) and facial veins (AFV) of the left side (∗).

A magnetic resonance imaging of the head and a neurological assessment were performed with negative results. In November 2014, she was brought to our attention to assess her intra- and extracranial venous vessels. ECD evaluation of neck veins in the supine position revealed atypical anterior jugular veins (AJVs) of increased size with normal valve leaflets in the proximal segment of the right vein and incontinence on the left side. The right AJV exhibited normal behavior during the Valsalva maneuver with temporary blocking of the outflow, whereas the left AJV revealed significant reflux from the confluence with the subclavian vein to the distal segment (J3) of the IJV through the anterior and common facial veins (AFV, CFV) (Figure 3; see Video 1 in Supplementary Material available online at http://dx.doi.org/10.1155/2015/960950).

**Figure 4:**
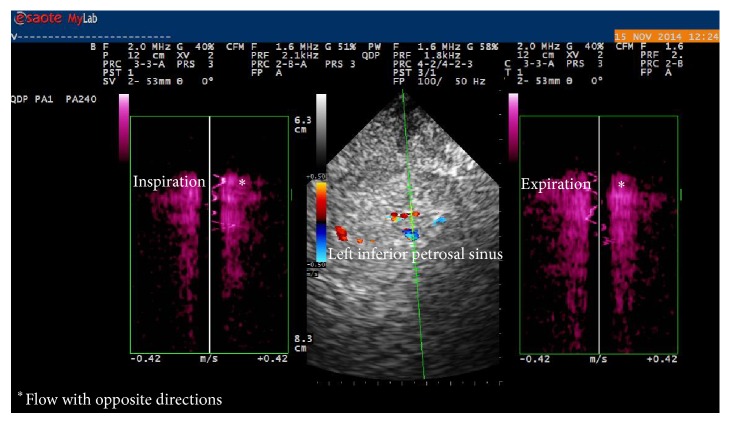
Reflux (∗) in the left inferior petrosal sinus.

The reflux also reached the superior ophthalmic vein (SOV) through the AFV and the nasofrontal vein. In the sitting position, we only observed the reflux during the Valsalva maneuver, involving the proximal segment of the left AJV. The transcranial approach through the supracondylar window revealed spontaneous reflux in the left inferior petrosal sinus in the supine and sitting positions (Figure 4).

## 3. Discussion

We believe this case report is interesting because it focuses on the potential role of chronic retinal venous congestion related to the impairment of extracranial outflow in the pathogenesis of CNV. To date, studies have focused on extrinsic mechanisms involved in CNV, such as the excessive elongation of the eyeball in patients with high myopia, thereby stretching the choroid, reducing arterial flow due to the increase in vascular resistance, and resulting in the release of vascular endothelial growth factor (VEGF) [3, 4]. Similarly, numerous researchers have revealed increased VEGF expression in human dural arteriovenous fistula specimens and in animal models of experimental venous hypertension [5, 6]. Our case appears to confirm these experimental data. In fact, the damage to the valve leaflets of the left AJV was likely caused by a block when swallowing. This damage produced a state of venous reflux not constant but variable with posture and respiration; retinal outflow is reduced in the supine position and primarily at night. Over time, these alternative pathways become overloaded by carrying their own draining flow and the shunted flow. In the light of this case, it could also be appropriate to investigate the chronic alterations of intra- and extracranial venous drainage as potential contributors to CNV in myopic patients.

## Supplementary Material

Left and right anterior jugular veins (AJV) during a normal breathing and at Valsalva manoeuvre.
The right vein shows a normal outflow with temporary blocked flow during the Valsalva. Along the left vein we see a spontaneous reflux during the normal breathing with a massive increase evoked from the Valsalva also interesting the homolateral anterior facial vein ( L AFV).

## Figures and Tables

**Table 1:** Ophthalmological examination.

Date of examination: January 23, 2013		

Patient	Gender	Birthdate

V. A.	Female	November 20, 1957

*Case History *		
Patient complains of acute onset of blurred vision in the left eye		

Best corrected vision		
Right eye	0.8 −5.25 sf −1.5 cyl @65°	
Left eye	0.4 −5.25 sf −1.25 cyl @120°	
Eye pressure		
Right eye	14 mmHg	
Left eye	14 mmHg	
Anterior segment		
Right eye	Nuclear cataract	
Left eye	Nuclear cataract	
Ophthalmoscopy		
Right eye	Normal	
Left eye	Macular hemorrhage	

*Conclusions *		
Clinical pictures compatible with CNV in pathological myopia (fluorescein angiography not performed because of allergy). We recommend intravitreal therapy with anti-VEGF drug		
